# Exploring physiotherapists’ clinical definition and diagnosis of inflammatory conditions of the lactating breast in Australia: a mixed methods study

**DOI:** 10.1186/s13006-020-00294-9

**Published:** 2020-05-24

**Authors:** Emma Heron, Tanya Maselli, Adelle McArdle, Beatriz I. R. de Oliveira, Leanda McKenna

**Affiliations:** 1 School of Physiotherapy and Exercise Science, Curtin University, GPO Box U1987, Perth, WA 6845 Australia; 2 Monash Rural Health Churchill, Monash University, Northways Road, Churchill, VIC 3842 Australia

**Keywords:** Females, Mastitis, Breast, Abscess, Lactation, Postpartum period

## Abstract

**Background:**

Differences in physiotherapy intervention practices for mastitis have been shown across Australian regions and facilities and it is unknown if this is associated with physiotherapists’ definition and diagnosis of Inflammatory Conditions of the Lactating Breast (ICLB). The aims were to determine how Australian physiotherapists’ define and diagnose ICLB and if there are regional or facility differences in their ICLB definition and diagnosis.

**Method:**

A cross-sectional mixed methods design was used to investigate how physiotherapists construct a definition and diagnosis of ICLB, via online qualitative and quantitative questions. Participants included 63 Australian physiotherapists who treated at least one woman with ICLB per month, over the last year. Thematic analysis and descriptive statistics were used to analyse qualitative and quantitative responses, respectively.

**Results:**

ICLB definition varied among physiotherapists (*n* = 63) with generated themes including definitions based on pathophysiology (57%), combination of local and systemic symptoms (38%), conditions (32%), local symptoms (25%) and breast function (16%). Overall, quantitative data supported these findings, as some physiotherapists considered blocked ducts an ICLB (83%), but some did not (17%), and some considered abscess and engorgement an ICLB (65%) and some did not (35%). For ICLB diagnosis, the main theme generated was lack of consensus between physiotherapists (*n* = 39) on the number or combination of local or systemic symptoms required. Quantitative data confirmed these themes, as 63% of physiotherapists (*n* = 63) indicated that more than one symptom was necessary to clinically diagnose ICLB, but 27% required only one symptom. For region and type of facility, consistency across the themes for region and facility was evident. Overall, quantitative data confirmed these findings, with no regional or facility differences, except physiotherapists from the Australian state of Victoria (96%) were more likely to consider blocked ducts as an ICLB, compared to those from the states of NSW (71%) or WA (71%) (*n* = 58; *χ*^**2**^ *= 6.49, p = 0.04*).

**Conclusion:**

Australian physiotherapists have varied definitions of ICLB and the required ICLB symptoms for clinical diagnosis. These results may prompt physiotherapists, who treat ICLB, to engage in explicit communication when discussing an ICLB in patient care, when delivering information in training courses and in developing treatment guidelines.

## Background

The definition of mastitis adopted by the Academy of Breastfeeding Medicine (ABM) is breast inflammation presenting with “a tender, hot, swollen, wedge-shaped area of breast associated with temperature of 38.5°C or greater, chills, flu-like aching, and systemic illness” [1](p.239), and is consistent with other definitions [2, 3]. Mastitis affects up to 20% of lactating women worldwide [4–11] with the highest incidence occuring in the first 6 months postpartum [12]. Untreated, mastitis can substantially impact on the mother’s health and daily functioning, potentially leading to the cessation of breastfeeding [1, 5]. Studies have shown that early cessation of breastfeeding reduces the known health benefits of lactation and breastfeeding to both mother and infant [13–16]. Thus it is important to support maintenance of breastfeeding and address potential barriers to continued breastfeeding, such as mastitis.

Mastitis is a narrow term meaning ‘breast inflammation’, from the latin ‘mastos’ (breast) and ‘itis’(inflammation) [17, 18]. However lactating breast conditions, such as breast abscess, blocked ducts or engorgement also present with clinical features of inflammation [19, 20], but may not be classified by physiotherapists as inflammatory. It can be difficult to differentiate between these inflammatory conditions based on clinical features alone [19, 20]. The ability of physiotherapists to consistently define and diagnose these inflammatory conditions may be problematic as there is little evidence to support the different diagnoses or to determine if these inflammatory conditions all have a similar causal pathway and natural history. As part of a healthcare team, physiotherapists provide care for women with lactating breast conditions, which may include therapeutic ultrasound, gentle massage, advice and education [21].

Contrastingly, Inflammatory Conditions of the Lactating Breast (ICLB) is an umbrella term that encompasses all inflammatory breast conditions. It is becoming more commonly used amongst physiotherapists since the introduction of a similar term in 2006 [22] and the use of the term in the only training course in lactation endorsed by the Australian Physiotherapy Association (APA). Utilising a term that highlights the concept of inflammation for these conditions may enhance patient care as physiotherapists currently use inflammatory symptoms to guide diagnosis and interventions. It is unknown whether this overarching diagnostic label is clinically appropriate. It is also unclear if physiotherapists would consider ICLB a suitable umbrella term for lactating breast conditions.

An audit of mastitis intervention practices demonstrated differences across Australian regions and facilities [21]. It was suspected that these differences might be related to how physiotherapists in different regions and facilities define and diagnose ICLBs. This would have implications for the transfer of care for patients between physiotherapists, regions and facilities and the development of national treatment guidelines. Therefore, the aims of this study were to determine how physiotherapists clinically define and diagnose ICLB and to determine if there are regional or facility differences in physiotherapists’ clinical ICLB definition and diagnosis.

## Methods

### Design

This was a cross-sectional mixed methods study exploring how Australian physiotherapists construct their clinical definition and diagnosis of ICLB. Quantitative and qualitative data was obtained via secure online questionnaire that asked both closed (pick list) and open-ended questions (Qualtrics, Provo, UT, USA). Demographic data was collected via nine closed-ended quantitative questions, followed by another three closed-ended and two open-ended qualitative questions that investigated ICLB definition and diagnosis (Table 1). Physiotherapists were asked which symptoms or combination of symptoms lead them to diagnose specific breast conditions that could potentially be considered an ICLB. Physiotherapists were also required to identify which conditions they considered an ICLB. This was the first set of questions from a larger questionnaire exploring physiotherapists’ rationale for choice of interventions for ICLB. The results are reported using the Standards for Reporting Qualitative Research [23].

**Table 1:** Questionnaire

Domains	Question summary	Question type
Demographics	1. Postcode of work facility where the majority of the physiotherapist’s ICLB caseload was treated	Closed: Text entry
	2. Number of full time years as a physiotherapist in clinical practice	Closed: Text entry
	3. Any post graduate qualifications in women’s health	Closed: Yes/No/Currently completing
	4. Type of post graduate qualifications in women’s health	Closed: Text entry
	Sub-question: Geographic location of post-graduate qualification	Closed: Text entry
	5. Any continuing education regarding the lactating breast	Closed: Yes/No/Currently completing
	6. Type of continuing education regarding the lactating breast	Closed: Text Entry
	7. Type of work facility where the majority of the physiotherapist’s ICLB caseload was treated	Closed: Five selected facility choices with “Other” text entry choice
	8. Type of referral to the physiotherapist for women with ICLB	Closed: Two selected referral choices with “Other” text entry choice
	9. Number of years of treating women with ICLB	Closed: < 2 years, 2–5 years, 6–10 years, > 10 years
Definition	10. Clinical definition of ICLB to another physiotherapist	Open: Text entry
	11. Conditions considered an ICLB – abscess, blocked ducts, engorgement, mastitis, other	Closed: Yes/No with “Other” text entry choice
Diagnosis	12. Symptoms or combination of symptoms that lead to diagnosis of the following conditions – abscess, blocked ducts, engorgement, mastitis, other	Closed: 17 selected symptom choices and two “Other” text entry choices
	13. Number of symptoms required for diagnosis of ICLB	Closed: Only one/More than one/Unsure
	14. Combination of symptoms considered important to diagnose ICLB	Open: Text entry

A pilot study that included five eligible physiotherapists, representing the major Australian population regions, was conducted from mid-July to early-August 2018, to ensure the questions were clearly understood, captured the required information and could be completed within 30–40 min. The pilot physiotherapists were colleagues of the researchers (EH, TM, NT, LM, AM) chosen based on their clinical experience treating ICLB. Alterations to the questions were based on email feedback from the pilot physiotherapists.

### Participants

Participants were physiotherapists who treated women with ICLB. Eligibility was determined through online screening via the questionnaire link. Three closed questions preceding the questionnaire confirmed physiotherapists met the inclusion criteria: an Australian registered and practicing physiotherapist, treating women with ICLB, with an average frequency of at least monthly over the past 12 months. Physiotherapists were recruited across all eight regions (states and territories), representing private practices and public (hospital) facilities.

A systematic approach for recruitment was used to obtain a representative sample of physiotherapists, as no database of Australian physiotherapists who treat ICLB exists. Each region was mapped to identify all facilities that would employ Australian women’s health physiotherapists (those who offer women’s health services). Mapping was achieved through cross referencing Department of Health and Continence Foundation of Australia websites, regional women’s health directories, the APA “Find a Physio” database and online searching for advertisements of women’s health physiotherapy services. Contact emails were sourced online or through direct telephone contact with the facility. Recruitment posters were placed on the national women’s health physiotherapy Facebook page, LinkedIn accounts and women’s health physiotherapy postgraduate university online forums. The APA was contacted to distribute the electronic link to members of the women’s, men’s and pelvic health special interest groups. Snowball recruitment was also utilised.

This study was conducted between mid-August and mid-November 2018. To maintain anonymity, all personal data was de-identified prior to analysis and only postcode information; a four-digit number that provides geographical representation of Australian regions, was used to indicate the geographical location of participants.

Information power for the qualitative component of this study was achieved through the establishment of specific aims, inclusion of physiotherapists with specialised knowledge, and clearly stated online quantitative and qualitative questions [24]. Information power was considered to be the amount of information obtained from women’s health physiotherapists that generated conceptually relevant themes [25]. Data provided by physiotherapists were continuously assessed to determine whether sufficient information was obtained to achieve saturation, but recruitment was stopped at the end of the advertised period.

### Data analysis

Data was exported into Microsoft Excel, then cleaned and checked for improbable answers and outliers (LM, BO). Quantitative data was used to verify and enhance the credibility of qualitative information via methodological triangulation [26]. Thus, the results from the quantitative data and the qualitative data were compared to determine if they generated similar information. Descriptive statistics, such as means, counts and percentage of responses, were generated for quantitative data, and counts and percentages were generated for qualitative data to estimate the frequency of each theme. To determine regional and facility differences, chi square, Fisher’s exact test or McNemar’s test was used, as appropriate.

NVivo 11 Software (QSR International, Melbourne, Australia) was used to organise the qualitative data to allow for a rigorous qualitative thematic analysis approach [27, 28]. Deductive thematic analysis was optimal for investigating physiotherapists’ clinical definition and diagnostic criteria of ICLB as it enabled the generation of a set of themes that best reflected the content of the data in light of the research aims, while inductive thematic analysis was also used to derive themes from the raw data that were not determined a priori. Thematic analysis involved six phases: data familiarisation, code generation, theme generation, theme revision, theme definition, and result reporting [27]. Three trained investigators (EH, BO, LM) independently conducted the analysis to improve the credibility of codes and themes. All coders were physiotherapists with experience in women’s health physiotherapy and women’s health physiotherapy research.

All coders read the written responses several times and labelled keywords with codes and grouped them into associated themes. Cross-coding was employed to develop a coding tree (Additional file 1) that was applied to all responses. Recurring themes were compared and discussed between coders to refine the main themes. The data was additionally inspected for differences and similarities of responses across regions and facilities (EH, BO, LM). Findings were further scrutinised by the extended research team to challenge and refine interpretations, ensuring robustness of the analysis.

## Results

### Flow of participants through the study

**Fig. 1:**
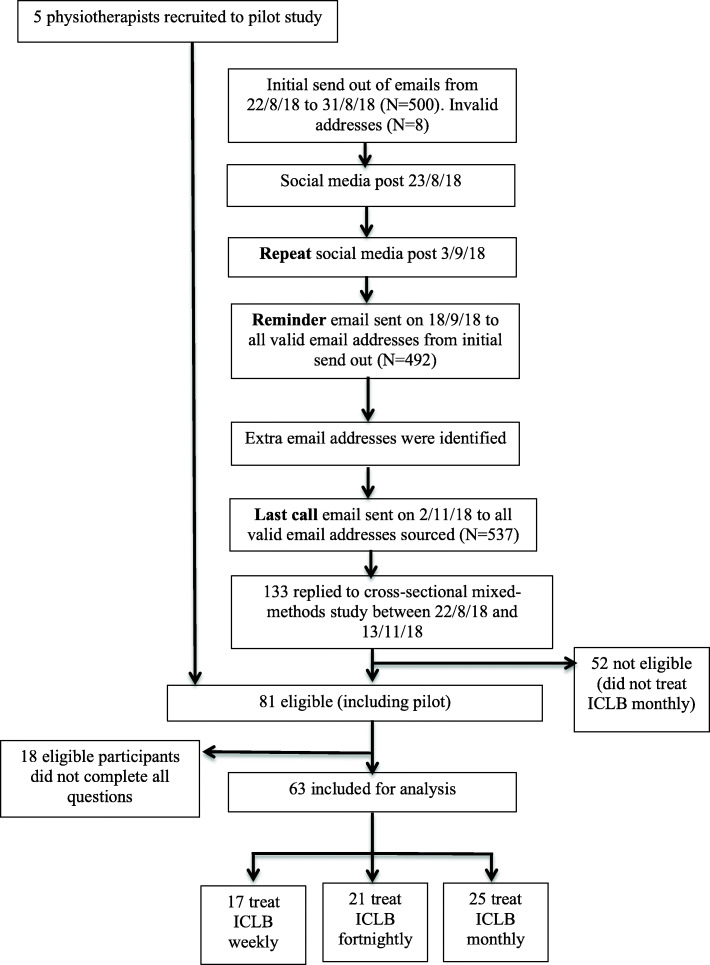
Flowchart of participant recruitment

A total of 537 valid email addresses of physiotherapists who could potentially meet eligibility criteria, were sourced from systematic nationwide recruitment strategies, and were sent the online link. Two reminder emails and one repeat social media post containing the online link were performed to maximise response rates, resulting in a total of 81 eligible physiotherapy participants (Fig. 1) [29].

Due to a small number of responses in some regions, only comparisons between NSW, Victoria and WA were analysed. Additionally, the small number of responses from some types of facilities (e.g. public health clinic) meant that only comparisons between private practices and hospital settings (either private or public) were conducted.

### Participant demographics

Most physiotherapists were located in Victoria, WA and NSW, had post-graduate qualifications, worked in private practice and had more than 2 years’ experience treating women with an ICLB (Table 2).

**Table 2:** Demographic characteristics of participants (*N* = 63)

	N (%)
**Years as a physiotherapist** (*n* = 63) (Mean (SD))	14.7 (9)
**Location** (*n* = 63)	
NSW/ACT	14 (22)
QLD	3 (5)
SA	1 (1.5)
TAS	1 (1.5)
VIC	27 (43)
WA	17 (27)
**Post-graduate qualification** (*n* = 63)	
No	12 (19)
Yes	49 (78)
Currently completing	2 (3)
**Type of post-graduate qualification** (*n* = 51)	
Short course	2 (4)
Post-graduate cert/graduate cert/professional cert	36 (70)
Post-graduate diploma of Women’s Health	1 (2)
Masters	12 (24)
**Location of post-graduate qualification** (*n* = 51)	
SA	1 (2)
VIC	25 (49)
WA	16 (31)
Overseas	1 (2)
Location of course not disclosed	8 (16)
**Continuing education in area of lactating breast** (*n* = 63)	
No	20 (31.7)
Yes	43 (68.3)
**Type of continuing education in area of lactating breast** (*n* = 43)	
In-service/conference/lecture	6 (14)
Short courses	32 (74)
Masters of Women’s Health	2 (5)
Format not described	3 (7)
**Place of work** (*n* = 63) (As per standard instruction, the data must be in a single paragraph; thus, the section was modified accordingly. Please check if appropriate).	
Private practice	41 (65)
Private hospital	2 (3)
Public hospital	15 (24)
Public health clinic	1 (1.6)
Private practice and private hospital	3 (4.8)
Public hospital and public health clinic	1 (1.6)
**Type of referral to the physiotherapist** (* n* = 63) (As per standard instruction, the data must be in a single paragraph; thus, the section was modified accordingly. Please check if appropriate).	
Primary practitioner	40 (63.5)
Referrals within MDT	16 (25.4)
Primary practitioner + MDT referral	6 (9.5)
Primary practitioner + maternal health nurse	1 (1.6)
**Years treating women with ICLB** (*n* = 63) (As per standard instruction, the data must be in a single paragraph; thus, the section was modified accordingly. Please check if appropriate).	
< 2 years	6 (10)
2–5 years	26 (41)
6–10 years	10 (16)
> 10 years	21 (33)

Note: *ACT* Australian Capital Territory; *ICLB* Inflammatory Conditions of the Lactating Breast; *MDT* Multi-Disciplinary Team; *NSW* New South Wales; *NT* Northern Territory; *QLD* Queensland; *SA* South Australia; *SD* Standard deviation; *TAS* Tasmania; *VIC* Victoria; *WA* Western Australia

### Primary outcome

#### Physiotherapists’ definition of ICLB

Physiotherapists (*n* = 63) provided a variety of definitions for ICLB. The themes and supporting quotes are summarised in Table 3. The main subthemes identified included local breast clinical symptoms, combination of local and systemic clinical symptoms, pathophysiology/cause, discrete breast conditions, and altered breast function (Additional file 2).

**Table 3:** Thematic responses of physiotherapists – clinical definition and diagnosis of ICLB

**Theme 1: The clinical definition of ICLB varies and may be based on its diagnostic symptoms and/or pathophysiology, or may include breast conditions**	
Subtheme 1.1: Definition of ICLB based on local and systemic clinical signs and symptoms presented by the patient	
*May consist of redness, increased temperature of affected area, tenderness on palpation, palpable lump, and fever or flu like symptoms (P1)*	
*Swelling, lump, pain and or redness over part of one or both breasts in the breast feeding woman (P45)*	
Subtheme 1.2: Definition of ICLB based on pathophysiology/cause	
*Pressure on milk ducts that has caused breast milk to leak into surrounding breast tissue and cause an inflammatory response. The body’s response to these “injuries” is usually quite extreme. (P4)*	
*When milk stasis occurs in a duct … chemicals unique to the milk can seep out of the semi permeable membrane of the duct into the parenchymal tissue. As this chemistry is foreign to the body outside the duct - an immediate, often severe inflammatory process is triggered (P2)*	
Subtheme 1.3: Definition of ICLB using breast conditions	
*‘Non-infective mastitis’ or ‘Blocked ducts’* i.e. *ICLB (P4)*	
*May include mastitis, milk stasis, breast abscess* etc. *(P92)*	
Subtheme 1.4: Definition of ICLB comprised of altered breast function	
*Inflammation of the breast tissue potentially resulting in impaired milk transfer (P55)*	
*Bothersome deep thickening (lump) of a section or sections of the lactating breast possibly associated with*. *.*. *reduced flow of milk from the affected breast (P87)*	
**Theme 2: The diagnosis of ICLB is based on the patient’s presenting signs and symptoms but there is no consensus on the specific number or combination of local symptoms required, or if it includes systemic symptoms**	
Subtheme 2.1: Diagnosis of ICLB based on the presence of local symptoms only	
*Breast lump (hard area) + either, tender, red, hot* (P2)	
*Pain or local tenderness, a degree of local tension/swelling* (P3)	
Subtheme 2.2: Diagnosis of ICLB based on the presence of local symptoms which is often associated with systemic clinical symptoms	
*Local symptoms including pain, redness, tension; global symptoms such as feeling unwell; plus some difficulty lactating (P4)*	
*Combines with subjective symptoms of feeling unwell, objective assessment needs to include redness, firmness or increased breast tension, and there must be a palpable breast tension/lump/firmness (P96)*	

*Theme 1: The clinical definition of ICLB varies among physiotherapists.*

*Subtheme 1.1: The definition of ICLB was based on the presence of local and systemic clinical symptoms.*

Many physiotherapists (*n* = 24, 38%) defined ICLB by the presence of a combination of local and systemic clinical symptoms: “*the breast is hot, red and tender and the woman often feels extremely unwell”* (P2). This was contrasted by 16 physiotherapists (25%) who based their definition on the presence of local clinical symptoms only: “*any condition causing signs of inflammation such as redness, pain, swelling, heat*” (P57).

*Subtheme 1.2: The definition of ICLB was based on pathophysiology/cause.*

Thirty-six physiotherapists (57%) stated there was a pathophysiology associated with the definition of ICLB. Many physiotherapists (*n* = 29, 46%) defined ICLB as an inflammatory process/response in the breast tissue with 14 physiotherapists (22%) stating it could have an infective or bacterial component/cause: *“either bacterial or non-bacterial cause of inflammation within breast tissue”* (P4). A total of 14 physiotherapists (22%) also included a physical or physiological cause of ICLB in their definition: “*most commonly caused by a change in routine with feeding or difficulties with emptying the breast with feeding because of issues with the baby latching, nipple issues or feeding positions*” (P81); “*protein in milk crosses the duct wall into the surrounding tissue and sets up a local inflammatory response*” (P20).

*Subtheme 1.3: The definition of ICLB was constructed around discrete breast conditions.*

In contrast, some physiotherapists (*n* = 20, 32%) listed discrete breast conditions: “*engorgement, blocked ducts, mastitis*” (P106) to define ICLB, with a few (*n* = 3, 5%) specifying it as a continuum of conditions, from *“.*. *. milk stasis – inflammation – blocked duct – mastitis*. *.*.” with potential for “*… serious progression to abscess and sepsis”* (P66).

*Subtheme 1.4: The definition of ICLB comprised of altered breast function.*

An additional 10 physiotherapists (16%) also included a disruption to feeding or breast function when defining ICLB: “*inflammation of the breast tissue during lactation … most commonly associated with reduced function of the breast*” (P49).

Quantitative data corroborated the themes of variability in definitions given for ICLB. Except for mastitis, physiotherapists did not unanimously agree on what conditions would be considered an ICLB (Table 4). For example, only 65% of physiotherapists considered abscess and engorgement an ICLB.

**Table 4:** Conditions considered an ICLB and number of symptoms required for ICLB diagnosis

Item	N (%)
**Condition considered an ICLB** (*N* = 63)	
Mastitis	63 (100)
Blocked ducts	52 (83)
Abscess	41 (65)
Engorgement	41 (65)
Milk bleb	5 (8)
Nipple trauma	2 (3)
Milk leeching	1 (1.5)
**Number of symptoms required for ICLB diagnosis** (*N* = 63)	
More than one symptom	40 (63)
One symptom	17 (27)
Unsure	6 (10)

Note: *ICLB* Inflammatory Conditions of the Lactating Breast

#### Physiotherapists’ diagnosis of ICLB

Physiotherapists (*n* = 39) provided a variety of combinations of clinical symptoms important for the diagnosis of ICLB. The themes and supporting quotes are summarised in Table 3. The main subthemes identified included local clinical symptoms and local and systemic clinical symptoms (Table 3). For these subthemes, generated themes included type and number of symptoms (Additional file 2).

*Theme 2: The diagnosis of ICLB is based on the patient’s presenting symptoms but there is no consensus on the specific number or combination of local symptoms required, or if it includes systemic symptoms.*

*Subtheme 2.1: The diagnosis of ICLB is based on the presence of local clinical symptoms only.*

Several physiotherapists (*n* = 22, 56%) believed a combination of local breast clinical symptoms (e.g. local pain/tenderness, tension/lump, erythema/redness, increased skin temperature/hot) to be important in diagnosing ICLB: “*pain, erythema, tension, increased skin temperature*” (P4).

*Subtheme 2.2: The diagnosis of ICLB is based on the presence of local symptoms which is often associated with systemic clinical symptoms.*

In contrast, 10 physiotherapists (26%) considered a combination of local symptoms plus the presence of systemic symptoms to be important in diagnosing ICLB “*Change to local breast tissue such as lump/swelling, colour change and pain which may also be associated with systemic change such as fever/chills/temp*” (P21).

Quantitative data corroborated the theme of inconsistency in the type of symptoms required to diagnose an ICLB. Although all physiotherapists believed that mastitis presents with both local inflammatory and systemic symptoms, there was less consistency in whether both local and systemic symptoms were required to diagnose the other ICLB conditions (Table 5).

**Table 5:** Diagnosis of specific breast conditions by symptoms (*N* = 63)

Symptoms	Breast conditions N (%)			
	Abscess	Blocked Duct	Engorgement	Mastitis
**Local**				
Breast pain	52 (83)^m^	56 (89)	58 (92)	60 (95)
Breast tenderness to touch	51 (81)^m^	57 (90)	58 (92)	60 (95)
Breast paraesthesia	28 (44)^b^	13 (21)^m^	20 (32)	26 (41)
Breast redness	48 (76)^bem^	37 (59)^m^	28 (44)	60 (95)^e^
Breast swelling	46 (73)^e^	40 (63)^em^	56 (89)	50 (79)^e^
Breast tension	40 (63)^e^	40 (63)^e^	54 (86)	47 (75)
Increase in local breast temperature	47 (75)^bem^	31 (49)^m^	33 (52)	58 (92)^e^
Breast lump	60 (95)^bem^	54 (86)^e^	20 (32)	49 (78)^e^
Milk bleb	4 (6)^bm^	28 (44)^em^	6 (10)	17 (27)^e^
Sore nipples including nipple vasospasm	8 (13)^bem^	19 (30)	20 (32)	22 (35)
Cracked nipples	9 (14)^m^	16 (25)^m^	11 (17)	25 (40)^e^
Enloculated^p^	2 (3)			
Orange peel^p^	2 (3)			
**Systemic**				
Flu-like symptoms	37 (59)^bem^	12 (19)^m^	6 (10)	59 (94)^e^
High temperature	40 (63)^bem^	9 (14)^m^	5 (8)	58 (92)^e^
Malaise	36 (57) ^bem^	9 (14)^m^	5 (8)	58 (92)^e^
Chills	37 (59) ^bem^	8 (13)^m^	4 (6)	56 (89)^e^
Headache	26 (41) ^bem^	8 (13)^m^	5 (8)	48 (76)^e^
Increased tiredness	31 (49)^bem^	13 (21)^m^	13 (21)	49 (78)^e^
Night sweats^p^	1 (2)			
Not resolving^p^	1 (2)			
History^p^	0 (0)	4 (6)	1 (2)	1 (2)

Note: McNemar’s test of significance was used. ^b =^ significantly different to blocked ducts; ^e =^ significantly different to engorgement; ^m =^ significantly different to mastitis; ^p^ symptom suggested by the physiotherapist

Qualitative responses from physiotherapists indicated that a variety of symptoms were needed to diagnose ICLB, and the most frequently listed were swelling/tension/lump (*n* = 27, 69%), followed by pain/tenderness (*n* = 24, 62%) and redness (*n* = 23, 59%). For number of symptoms, a proportion of physiotherapists (*n* = 14, 36%) thought that three local symptoms were needed to diagnose ICLB: “*at least 3 symptoms i.e. Pain, swelling and redness or increased temperature*” (P94), closely followed by two local symptoms (*n* = 13, 33%): “*unilateral pain and swelling are the main 2 symptoms I look for*” (P20).

Quantitative data corroborated the theme of inconsistency in the specific number of symptoms required to diagnose ICLB. Almost two thirds of physiotherapists indicated that more than one symptom was necessary to make a clinical diagnosis of ICLB (Table 4). Most of the remaining physiotherapists required the presence of only one symptom (Table 4).

#### Regional and facility differences

There was consistency in the variety of qualitative themes identified across regions. Quantitative data only revealed a significant difference (*N* = 58, *χ*^2^ *= 6.49, p = 0.04*) between physiotherapists from different regions for whether or not blocked ducts were considered an ICLB. Physiotherapists from Victoria (*n* = 26; 96%) were more likely to consider that blocked ducts are an ICLB, compared to NSW (*n* = 10; 71%) or WA (*n* = 12; 71%). No other differences between regions existed for the remaining quantitative data.

Consistency was also observed in the qualitative themes regardless of the facility in which the physiotherapist works. No differences were found between hospital and private practice physiotherapists for the quantitative findings.

## Discussion

Overall, qualitative results emphasised variance amongst physiotherapists’ clinical definition of ICLB and symptoms required for diagnosis. Quantitative results indicated that abscess, blocked ducts, engorgement and mastitis were the main conditions physiotherapists considered an ICLB. All physiotherapists considered mastitis an ICLB, but not all physiotherapists considered other breast conditions to be an ICLB. Overall, there was no difference between regions or facilities in physiotherapists’ definition and diagnosis of ICLB.

Variability amongst physiotherapists for the clinical ICLB definition could indicate that the ICLB term is not well recognised. Variations in the mother’s presenting symptoms may impact on how a physiotherapist defines ICLB. When considering how physiotherapists may theoretically construct ICLB and mastitis, only 38% defined ICLB in a similar manner to previous definitions of mastitis [1–3], by including themes of local and systemic signs and symptoms. Physiotherapists defined ICLB with broader constructs (Table 4), indicating that ICLB could be considered a wider umbrella term than mastitis alone. In addition, 100% of physiotherapists defined mastitis as an ICLB, which allows mastitis to be considered a subgroup of the umbrella term of ICLB.

The variability regarding diagnosis (whether systemic symptoms and the number of symptoms are required), could again be due to variations in the mother’s presenting symptoms. Additionally, other diagnostic criteria such as the mother’s rating of severity of symptoms or functional impact [30] associated with ICLB may need to be considered as part of the diagnosis of ICLB. When considering what symptoms physiotherapists used for diagnosis, there was greater consistency surrounding the diagnosis of mastitis, compared to abscess, blocked ducts, engorgement (Table 5). Symptoms named in the ABM definition [1] of mastitis were the most commonly used by physiotherapists to diagnose mastitis. Previously published guidelines do not explicitly state symptoms associated with engorgement [31] and a published guideline that states symptoms associated with clinical diagnosis in abscess, blocked ducts, and engorgement for health care practitioners is needed.

Physiotherapists have traditionally used cardinal signs and symptoms of inflammation (heat, redness, swelling, pain, loss of function) [32] to diagnose ICLB, and this is likely to influence intervention choices. Nearly all physiotherapists in this study used the cardinal symptoms of inflammation [32] to diagnose mastitis (Table 5), but not for abscess, blocked ducts or engorgement. Physiotherapists did not necessarily consider these conditions to be an ICLB (Tables 4 and 5), and the word ‘inflammatory’ within the label ICLB may need to be reviewed. Alternatively, the pathophysiology for abscess, blocked ducts and engorgement may need to be more evident, to support their inclusion under the ICLB label.

The identified variability amongst physiotherapists’ definition and diagnosis of ICLB was not associated with the geographic region or type of work facility. These results are contrary to the findings of a recent audit [21] that showed regional and facility differences in choice of intervention and parameters used in the treatment of mastitis. The current study indicates that these regional and facility differences may not be explained by the physiotherapist’s underlying definition or diagnostic criteria for mastitis and other factors need to be considered. The previous audit [21] examined what physiotherapists recorded in case notes, but the current study directly questioned physiotherapists, which may account for differences between studies. The only finding that indicated a difference in regions for definition or diagnosis, was that physiotherapists’ from the state of Victoria were more likely to include blocked ducts in their definition of ICLB. The reason for this is unclear, but may be related to post-natal education of breast care or clinical pathways that are specific to the medical management of blocked ducts in Victoria, compared to other states.

Physiotherapists need to be aware that not all of their clinical peers may view abscess, blocked ducts and engorgement as they do, and this has implications for intervention choices and communication when transferring patient care. Continuing education and future clinical guidelines would need to clarify ICLB definitions and diagnosis. Physiotherapists need to communicate clear concise information to their patients, to provide cohesive care. There is a need for an empirically defined overarching definition of ICLB, potentially achieved through a multidisciplinary, international Delphi expert panel [33]^.^ This could provide guidance to physiotherapists and may reflect the diverse symptoms that present to physiotherapists clinically.

Limitations of this study may include a response bias if physiotherapists responded with an “ideal” answer. While an online mode enhanced accessibility, individual interviews or focus groups could have rendered further information to investigate any differences between an ‘ideal’ versus the ‘clinically used’ definition of ICLB. This study surveyed only physiotherapists, and the viewpoints of other health care professionals have not been represented. Although generalisability of qualitative findings are limited, the robust sample size and strategic distribution of responses Australia-wide remains a strength of this study.

## Conclusions

This study showed that physiotherapists have varied impressions of what constitutes an ICLB and its diagnostic criteria. Clinically, this highlights the need for physiotherapists to clearly articulate their definition and diagnosis of an ICLB to patients and colleagues, for example when transferring care, and to improve treatment continuity. This study also indicates that continuing education courses, and future clinical guidelines would need to clarify definitions and diagnosis of ICLB. Overall, this study should prompt physiotherapists, who treat ICLB, to engage in explicit communication when discussing an ICLB.

## Supplementary information

**Additional file 1.** Microsoft word document (.doc); Coding tree – the codes generated from the data/physiotherapist’s responses for use in the thematic analysis.

**Additional file 2.** Microsoft word document (.doc); Coding manual – results of the thematic analysis including theme descriptions and examples.

## Data Availability

The datasets generated and/or analysed during the current study are not publicly available due to individual compromise of privacy but are available from the corresponding author on reasonable request.

## Abbreviations

ABM: Academy of breastfeeding medicine
APA: Australian physiotherapy association
ICLB: Inflammatory conditions of the lactating breast
