# Supplementary material for: Exploring physiotherapists’ clinical definition and diagnosis of inflammatory conditions of the lactating breast in Australia: a mixed methods study
Source: Int Breastfeed J. 2020 May 24;15:48. doi: 10.1186/s13006-020-00294-9 (PMC7247145; doi:10.1186/s13006-020-00294-9)
Supplement: Supplementary file 1 — Additional file 1. Microsoft word document (.doc); Coding tree – the codes generated from the data/physiotherapist’s responses for use in the thematic analysis. [file 13006_2020_294_MOESM1_ESM.docx]

# Additional File 1

## Coding tree

### Codes:

### Question 15: Definition of ICLB

- Local clinical symptoms only (n=16, 25%). Example codes: Heat/hot/increased temperature, pain/tenderness, redness/erythema, swelling/tension/lump.
- Combination of local and systemic clinical symptoms (n=24, 38%). Example codes: Same as above, plus: Aches, fever, flu like symptoms, malaise
- Pathophysiology/cause (n=36, 57%):
  - Inflammatory process/response (n=29, 46%). Example codes: Inflammation of/in the breast tissue
    - Infective/bacterial component/cause (n=14, 22%)
  - Physical/physiological cause (n=14, 22%). Example codes: Milk seeping into surrounding tissue, compression
- Discrete breast conditions (n=20, 32%). Example codes: Abscess, blocked ducts, engorgement, mastitis
  - Spectrum/continuum (n=3, 5%). Example codes: Milk stasis – inflammation – blocked duct – mastitis – abscess – sepsis
- Function (n=10, 16%). Example codes: Impaired milk transfer/feeding, reduced flow of milk

### Question 19: Diagnosis of ICLB

- Combination of local breast clinical symptoms (n=22, 56%). Example codes:
  - Type of symptoms:
    - Swelling/tension/lump (n=27, 69%)
    - Pain/tenderness (n=24, 62%)
    - Redness (n=23, 59%)
    - Heat/warmth (n=11, 28%)
    - Altered function (n=7, 18%)
  - Number of symptoms:
    - One local symptom (n=2, 5%)
    - Two local symptoms (n=13, 33%)
    - Three local symptoms (n=14, 36%)
    - Four or more local symptoms (n=7, 18%)
- Combination of local and often systemic symptoms (n=10, 26%). Example codes: Same as above, plus: flu like symptoms, unwell.

### Table 6

Coding tree

| **Overarching theme** | **Codes** | **Example codes** |
| --- | --- | --- |
| Q15 Definition of ICLB | Local symptoms | Pain/tenderness, redness/erythema, swelling/tension/lump, heat/hot/increased temperature |
|  | Combination of local and systemic symptoms | Fever, flu like symptoms, aches, malaise |
|  | Pathophysiology/cause |  |
|  | Inflammatory process/response | Inflammation of/in the breast tissue |
|  | Physiological or physical cause | Milk seeping into surrounding tissue, compression |
|  | Discrete conditions | Mastitis, blocked ducts, engorgement, abscess |
|  | Spectrum/continuum | Milk stasis – inflammation – blocked duct – mastitis – abscess – sepsis |
|  | Function | Impaired milk transfer/feeding, reduced flow of milk |
| Q19 Diagnosis of ICLB | Combination of local symptoms |  |
|  | Type of symptoms | Swelling/tension/lump, pain/tenderness, redness, heat/warmth, altered function |
|  | Number of symptoms | One local symptom, two local symptoms, three local symptoms, four or more local symptoms |
|  | Combination of local and systemic symptoms |  |
