# Supplementary material for: Exploring physiotherapists’ clinical definition and diagnosis of inflammatory conditions of the lactating breast in Australia: a mixed methods study
Source: Int Breastfeed J. 2020 May 24;15:48. doi: 10.1186/s13006-020-00294-9 (PMC7247145; doi:10.1186/s13006-020-00294-9)
Supplement: Supplementary file 2 — Additional file 2. Microsoft word document (.doc); Coding manual – results of the thematic analysis including theme descriptions and examples. [file 13006_2020_294_MOESM2_ESM.docx]

# **Additional File 2**

## Coding manual

### Themes:

### Table 7

Question 15: Definition of ICLB (n=63)

| Theme title | Theme description | Example quote of theme |
| --- | --- | --- |
| Combination of local and systemic symptoms | ICLB was defined by the presence of a combination of local and often systemic clinical signs and symptoms by 24 physiotherapists (38%): “*The breast is hot, red and tender and the women often feels extremely unwell*” (P2). | “*May consist of redness, increased temperature of affected area, tenderness on palpation, palpable lump, and fever or flu like symptoms*” *(P1).*  “*Any inflammatory condition of the Lactating Breast that causes localised and/or general systemic infection symptoms ie. localised breast tenderness, firmness, redness and with/without systemic headaches, malaise, aches and fever*” *(P70).* |
| Local symptoms | Many physiotherapists (n=16, 25%) based their definition of ICLB on the presence of local breast clinical signs and symptoms: “*any condition causing signs of inflammation such as redness, pain, swelling, heat*” (P57). | “*Inflammation of the breast resulting in swelling, redness, heat, pain that can interfere with breastfeeding*” *(P124)*.  “*Swelling, lump, pain and or redness over part of one or both breasts in the breastfeeding woman*” *(P45)*. |
| Pathophysiology/cause  Inflammatory process/response  Physical cause (eg. Trauma) or physiological cause (eg. Milk in interstitium) | Thirty-six physiotherapists (57%) stated there was a pathophysiology associated with the definition of ICLB.  Many physiotherapists (n=29, 46%) defined ICLB as an inflammatory process/response in the breast tissue. 14 physiotherapists (22%) stated it could have an infective or bacterial component/cause: *“either bacterial or non-bacterial cause of inflammation within breast tissue”* (P4).  A total of 14 physiotherapists (22%) included a physical or physiological cause of ICLB in their definition: “*most commonly caused by a change in routine with feeding or difficulties with emptying the breast with feeding because of issues with the baby latching, nipple issues or feeding positions*” (P81); “*protein in milk crosses the duct wall into the surrounding tissue and sets up a local inflammatory response*” (P20). | *“When milk stasis occurs in a duct… chemicals unique to the milk can seep out of the semi permeable membrane of the duct into the parenchymal tissue. As this chemistry is foreign to the body outside the duct - an immediate, often severe inflammatory process is triggered (P2)”.*  “*In some presentations engorgement of milk in ductal tissue can result in milk seeping into surrounding tissue - triggering an inflammatory response. In other cases lymphatic vessels carrying lymph fluid towards lymph nodes in the underarm - may be compressed/restricted by engorged milk ductal tissue, compressive clothing, posture etc and can result in an insufficient removal of bacteria/pathogens*” *(P31).* |
| Discrete conditions  Spectrum/continuum | Some physiotherapists (n=20, 32%) listed discrete conditions: “*engorgement, blocked ducts, mastitis*” (P106) to define ICLB with:  A few (n=3, 5%) specifying it as a continuum of conditions, from “*…milk stasis – inflammation – blocked duct – mastitis…*” with potential for “*…serious progression to abscess and sepsis*” (P66). | *“‘Non-infective mastitis’ or ‘Blocked ducts’ ie. ICLB” (P4)*  *“May include mastitis, milk stasis, breast abscess etc.” (P92).* |
| Function | A few physiotherapists (n=10, 16%) included a disruption to feeding or breast function when defining ICLB: “*Inflammation of the breast tissue during lactation… most commonly associated with reduced function of the breast*” (P49). | “*Inflammation of the breast tissue potentially resulting in impaired milk transfer*” *(P55).*  “*Bothersome deep thickening (lump) of a section or sections of the lactating breast possibly associated with… reduced flow of milk from the affected breast*” *(P87).* |

### Table 8

Question 19: Diagnosis of ICLB (n=39)

| Theme title | Theme description | Example quote of theme |
| --- | --- | --- |
| Combination of local symptoms  Type of symptoms  Number of symptoms | Many physiotherapists (n=22, 56%) believe a combination of local breast clinical signs and symptoms is important in diagnosing ICLB: “*pain, erythema, tension, increased skin temperature*” (P4).  Swelling/tension/lump (n=27, 69%), followed by pain/tenderness (n=24, 62%) and redness (n=23, 59%) were the most frequently listed local symptoms: "*pain, erythema, tension, increased skin temperature*" (P4).  Many physiotherapists (n=14, 36%) believe that three local symptoms are needed to diagnose ICLB: “*At least 3 symptoms ie pain, swelling and redness or increased temperature*” (P94), closely followed by two local symptoms (n=13, 33%): “*unilateral pain and swelling are the main 2 symptoms I look for*” (P20). | “*Breast lump (hard area) + either, tender, red, hot…*” *(P2).*  “*Pain or local tenderness, a degree of local tension/swelling*” *(P3).* |
| Combination of local and systemic symptoms | A combination of local symptoms plus often the presence of systemic systems is considered important in diagnosing ICLB by 10 (26%) physiotherapists: “*Change to local breast tissue such as lump/swelling, colour change and pain which may also be associated with systemic change such as fever/chills/temp*” (P21). | "*Local symptoms including pain, redness, tension; global symptoms such as feeling unwell; plus some difficulty lactating…"* *(P5).*  “*Combines with subjective symptoms of feeling unwell, objective assessment needs to include redness, firmness Or increased breast tension, and there must be a palpable breast tension/lump/firmness*” *(P96).* |
